# Acetic Acid Fermentation of Soybean Molasses and Characterisation of the Produced Vinegar

**DOI:** 10.17113/ftb.58.01.20.6292

**Published:** 2020-03

**Authors:** Lucas Caldeirão Rodrigues Miranda, Rodrigo José Gomes, José Marcos Gontijo Mandarino, Elza Iouko Ida, Wilma Aparecida Spinosa

**Affiliations:** 1 Department of Food Science and Technology, Londrina State University, Celso Garcia Cid (PR 445) Road, 86057-970, Londrina, PR, Brazil; 2 Embrapa Soybean, Carlos João Strass Road, 86001-970, Londrina, PR, Brazil

**Keywords:** vinegar, acetic acid fermentation, soybean molasses, by-product of soybean processing

## Abstract

Soybean molasses is a by-product from the production of protein concentrate from soybean meal that predominantly contains sugars, with sucrose as the major component. In Brazil, soybean molasses is used for animal feed or it is discarded, although some industries use it to produce ethanol. This study aims to evaluate the parameters required for the acetic acid fermentation of soybean molasses, and characterise the resultant vinegar. To study the most suitable parameters for the acetic acid fermentation, vinegar was produced from the alcohol fermentation of soybean molasses through eight fermentation cycles: five for adaptation and three for production. The average acidity of the acetic acid fermentation product was 50.60 g/L, with an acetic acid fermentation yield, total yield of acetic acid in broth and productivity 65.01%, 92.76% and 0.033 g/(L·h), respectively. The vinegar produced from soybean molasses has an acidity of 5.07% (*m*/*V*), residual ethanol content 0.17% (*m*/*V*), sugars 7.86% (*m*/*V*), dry extract 14.67% (*m*/*V*), ash 2.27% (*m*/*V*) and a density of 1.023 g/cm^3^. The contents of total phenolics and isoflavones decreased after the alcohol and acetic acid fermentations. Moreover, the isoflavones profile of the fermented product comprised only three forms: daidzein, glycitin and genistin. According to our results, 3460 L of vinegar can be produced for every tonne of soy molasses, with an acetic acid concentration of 40 g/L, the minimum required by the legislation on vinegar production. Thus, these findings demonstrate that soy molasses represents a useful raw material for the production of vinegar.

## INTRODUCTION

Soybean molasses is a viscous, brown and bittersweet liquid, obtained from the processing of soy protein concentrates. The soy proteins are concentrated from defatted soybean meal by ethanol washing, a procedure that promotes the extraction of soluble carbohydrates and other water-soluble compounds. Sugars are then concentrated *via* the evaporation of ethanol and the partial evaporation of water, resulting in the production of soybean molasses (*1*). Sucrose, raffinose and stachyose are the main sugars in the molasses, which also contains proteins, lipids, minerals and isoflavones (*1*, *2*). A large portion of this by-product is used for animal feed or discarded. In Brazil, however, some soybean processing industries use soybean molasses to produce hydrated alcohol, because sucrose is the predominant sugar and it can be fully metabolised by the yeast *Saccharomyces cerevisiae*. In contrast, raffinose and stachyose cannot be metabolised by *S*. *cerevisiae*, as it lacks the α-galactosidase enzyme necessary to hydrolyse the α-1,6-glycosidic bonds of these sugars (*1*, *3*).

The isoflavones are the main phenolic compounds present in soybean and its products. These compounds are distributed in aglycone forms (daidzein, genistein and glycitein) and their respective β-glucoside (daidzin, genistin and glycitin), acetyl-glucoside (6"-*O*-acetyl-daidzin, 6"-*O*-acetyl-genistin and 6"-*O*-acetyl-glycitin) and malonyl-glucoside conjugates (6"-*O*-malonyl-daidzin, 6"-*O*-malonyl-genistin and 6”-*O*-malonyl-glycitin), totalling twelve different forms (*4*). According to Handa *et al*. (*5*), the glycosidic forms are predominant in soybeans, constituting 50–90% of the total isoflavones. It is reported that these isoflavones, mainly the aglycones, have biological activity. Moreover, they have attracted attention because of their ability to reduce the risk of cardiovascular diseases, inhibit cancer cell growth, prevent diseases, such as osteoporosis, and alleviate the symptoms of menopause (*5*).

Vinegar is a food condiment produced by a double fermentation: an initial alcohol fermentation, followed by an acetic acid fermentation, for which amylaceous or sugary raw materials can be used (*6*–*9*). In biochemical terms, acetification is an oxidation process carried out by acetic acid bacteria (AAB). However, the process of vinegar production is commonly referred to as a fermentation and it can be performed in three different ways: slow, fast and submerged (*8*, *10*). The submerged fermentation consists of a controlled agitation and aeration of the must in which the obligate aerobic bacteria oxidise ethanol into acetaldehyde and then into acetic acid.

The process of acetic acid fermentation involves several bacterial genera, such as *Acetobacter*, *Gluconobacter*, *Gluconacetobacter* and *Komagataeibacter* (*10*, *11*). In an *ad-hoc* designed fermentor, the combination of agitation and aeration promotes the formation of micro-bubbles of air that facilitate the diffusion of oxygen, which increases the contact surface between the bacteria and the substrate, in turn, boosting the efficiency and productivity of the fermentation. The absence of aeration for even 1 min during this process can result in eight consecutive non-productive days of fermentation (*12*).

In the production of vinegar with a high content of acetic acid, the fermentation is typically a semi-continuous process, which operates in cycles. The cycle starts with a total volume fraction of broth ranging from 12 to 15% (7–10% acetic acid and 5% ethanol). When the ethanol volume fraction is between 0.2 and 0.3%, a relative volume of fermented broth is withdrawn, and a new volume is added to the medium (*13*).

In order to expand the use of soybean molasses, this work aims to identify the best parameters for the cycles of acetic acid fermentation of soybean molasses by the submerged semi-continuous process and, secondly, to characterise the obtained vinegar.

## MATERIALS AND METHODS

### Material

The ethanol from soybean molasses used for the production of vinegar was obtained in a previous work (*1*). It had an alcohol content of 6.00% (*V*/*V*) and acidity of 7.40 g/L (acetic acid). Isoflavone standards were obtained from Wako Pure Chemical Industries, Ltd. (Osaka, Japan) and Sigma–Aldrich Co. (St. Louis, MO, USA). All other reagents were of analytical grade and sourced commercially from various manufacturers as stated in the following chapters.

### Inoculum activation and preparation

Pure cultures of acetic acid bacteria (AAB) were not used for the production of vinegar in this work because the environmental conditions, such as low pH, presence of ethanol, aeration and temperature, naturally select the best producer species. The mixed culture of AAB was obtained during the production of unfiltered white vinegar, according to Spinosa (*14*) with modifications. This material was quickly collected from the German fermentor of a vinegar-producing industry (Tecnologia em Saúde, Assis, Brazil). This fermentor had a total capacity of 300 L and operated at 30 °C with 1.0% (*V*/*V*) ethanol and 40% (*m*/*V*) total titratable acidity. To obtain the initial inoculum, 50 mL of white vinegar were added to a flask containing 250 mL of mannitol–yeast broth (HiMedia, Mumbai, India) and placed in a shaking incubator (CT-712 R; Cientec, Belo Horizonte, Brazil) at 30 °C for 72 h. This initial inoculum was transferred to a bioreactor holding vessel containing 4 L of a broth prepared with a mixture of 2 L of unfiltered white vinegar containing 10.0% acetic acid (*m*/*V*) and 0.4 L ethanol with 50.0% alcohol (*V*/*V*), which contained a bacterial count equal to or greater than 8 log CFU/mL.

### Acetic acid fermentation

The bioreactor (6 L total volume) used for soybean molasses vinegar production in submerged fermentation at the pilot scale was obtained in a biorreactor (Rubia Basic; Biofoco, Piracicaba, Brazil). The conditions of the fermentation process in the bioreactor were (30±0.5) °C, agitation 800 rpm, aeration flow 0.50 L/min and aeration flow through culture medium 0.25 L/(mL∙min). During the process, the total acidity, alcohol content and aeration were adjusted, as the acidity and alcohol content are the determining parameters for loading and unloading the fermentation cycles. The total volume fraction of the initial broth was defined as the sum of the volume fractions of ethanol and acetic acid (in %, *m*/*V*). Therefore, the total volume fraction of the initial broth was 10% (5% (*V*/*V*) ethanol and 5% (*m*/*V*) acetic acid). The cycles of submerged acetic acid fermentation were conducted as a fed-batch and each one of these cycles is considered as a replicate of the experiment. When the alcohol content reached 0.5% (*V*/*V*), an aliquot of the vinegar volume was withdrawn from the reactor and the same volume of ethanol from soybean molasses was added.

### Acetic acid fermentation of ethanol from soybean molasses

After the conditions had stabilised, 0.680 L ethanol obtained from alcoholic fermentation of soybean molasses previously centrifuged (model 5804R; Eppendorf, Hamburg, Germany) at 12 000×*g* and 4 °C for 15 min was added to the bioreactor. Thus, the initial content of ethanol reached 1.52% (*V*/*V*).

Once the fermentation cycles had started, the vinegar was collected when the ethanol volume fraction in the vinegar reached *φ*≤0.5% (*V*/*V*). In order to maintain the total volume fraction of the broth, an adequate volume of the ethanol had to be replaced. To evaluate the most suitable parameters for the acetic acid fermentation of ethanol from soybean molasses, eight fermentation cycles were performed. The first five cycles were performed to facilitate culture adaptation, whereas during the last three cycles, the reactor only contained the acetic acid and the alcohol-fermented soybean molasses. For each cycle, the following parameters were determined: acetic acid fermentation yield (*Y*_AA_), total yield of acetic acid in the broth (*Y*_AA_(broth)) and productivity of acetic acid (*P*_AA_). These parameters were calculated using the following equations, respectively:

*Y*_AA_=*γ*_f_∙0.77∙100/*V*_R_ /1/

*Y*_AA_(broth)=*φ*_AAf_∙100/*φ*_AAi_ /2/

*P*_AA_=(*V*_f_∙*γ*_f_/*t*∙*V*_R_)∙10 /3/

where *γ*_f_ is the final acetic acid concentration (g/100 mL), 0.77 is the stoichiometric factor for conversion of ethanol to acetic acid, *V*_R_ is the volume of the reactor, *φ*_AAi_ and *φ*_AAf_ are the initial and final total volume fraction of acetic acid in the broth at the beginning and the end of the cycle (%), *V*_f_ is the volume (L) of removed vinegar and *t* is the time (h) of fermentation cycle.

### Characterisation of soybean molasses vinegar

The produced soybean molasses vinegar was distilled using an alcohol micro-distiller (TE-012; Tecnal, Piracicaba, Brazil), and the dry extract and ash contents were determined gravimetrically using an oven (Odontobrás EL 1.5; Ribeirão Preto, Brazil) at 105 °C and a muffle (Quimis, Diadema, Brazil) at 550 °C, respectively. The acidity of the distilled vinegar was determined by titration with 0.1 M NaOH (Química Moderna, Barueri, Brazil), using 1.0% phenolphthalein (Inlab, Diadema, Brazil) as an indicator, and it was expressed in g acetic acid per 100 mL solution. The ethanol content and relative density of the distilled vinegar were determined using a digital density meter (DDM 2909; Rudolph Research Analytical, Hackettstown, NJ, USA) at 20 °C and expressed in mL/L and g/cm^3^ respectively.

Total sugar content of the vinegar was determined according to DuBois *et al*. (*15*) in a spectrophotometer (Spectronic Genesys 6; Thermo Electronic Corporation, Waltham, MA, USA) at 490 nm using glucose (Synth, Diadema, Brazil) as standard. Total phenolic content was determined in a spectrophotometer (Spectronic Genesys 6; Thermo Electronic Corporation) at 760 nm with Folin-Ciocalteu reagent (Sigma-Aldrich Co., Merck, St. Louis, MO, USA). The results were expressed in mg gallic acid (Sigma-Aldrich Co., Merck) equivalents per 100 mL of the sample, as described by Singleton *et al*. (*16*).

To separate the different isoflavone forms present in the vinegar, we used a 1:1:1 volume ratio of ultrapure water (Merck Millipore, Burlington, MA, USA), acetone (Anidrol, Diadema, Brazil) and ethanol (Anidrol). The extracts were homogenized every 15 min for 1 h on a tube shaker (CT-712 R, Cientec), followed by sonication (Solidsteel, Piracicaba, Brazil) for 15 min and centrifugation (model 5804R; Eppendorf) at 794×*g* and 4 °C for 15 min. The supernatants were filtered using a 0.22-μm polyvinylidene fluoride (PVDF) membrane (Millex^®^, Millipore, Merck, Sigma-Aldrich Ireland Ltd., Arklow, Ireland) and analyzed by ultra-performance liquid chromatography (Acquity UPLC System, Waters, Milford, MA, USA), as described by Handa *et al.* (*5*).

### Cell count

The AAB used in the soybean molasses vinegar production were quantified by plate counting after allowing them to grow on a double layer of mannitol, yeast and peptone agar (HiMedia) (0.50 and 1.00% agar in the lower and upper layer, respectively) at 30 °C for 48 h. The counts were expressed in CFU/mL (*14*).

### Statistical analysis

Three independent assays were performed, and the average values were determined. Data are expressed as mean value±standard deviation using the statistical software R v. 3.5.3 (*17*).

## RESULTS AND DISCUSSION

### Evaluation of the parameters used for the acetic acid fermentation of ethanol from soybean molasses

The conversion rate of acetic acid (Table 1) decreased with the addition of the alcohol fermentation product from soybean molasses into the fermentor. The same was also observed for the productivity (*P*_AA_), which decreased considerably after the fourth cycle, starting with 0.106 g/(L·h) in the first cycle and decreasing to 0.023 g/(L·h) in the final cycle. This decrease can be related to the fact that soybean molasses ethanol contained considerable amounts of residual sugars (Table 2). In some AAB species, two main metabolic pathways are present: the pentose phosphate cycle for carbohydrate oxidation and the tricarboxylic acid cycle for the oxidation of organic acids (*18*). Due to the considerable amount of sugars in soybean molasses ethanol, a change in the metabolic pathway by the AAB might have occurred, which affected the productivity results. In this case, the microbiota could have prioritised the carbohydrate metabolism pathway, producing less acetic acid (product of ethanol metabolism) and more carbohydrate metabolism products, such as gluconic acid or exopolysaccharides (cellulose, levan, dextran or acetan) (*19*). In the conversion of ethanol into acetic acid, various intermediate compounds, including acetaldehyde, ethyl acetate and other alcohols, esters and acids, are also formed. Their nature and quantity depend on the characteristics of the raw material (wine) and they are responsible for the flavour of the vinegar (*14*).

**Table 1:** Cycles and parameters of acetic fermentation of soybean molasses

Cycle	*t/*h	*V*_B_/L	*φ*(ethanol)_i_/%	*γ*_i_ /(g/L)	*φ*_AAi_ /%	*V*_A_ /(L)	V_R_ /(L)	*γ*_f_ /(g/L)	*φ*(ethanol)_f_/%	*φ*_AAf_ /%	*Y*_AA_ /%		*Y*_AA_(broth)/%	*P*_AA_/(g/L·h)
1	49	4.30	1.52	56.20	7.14	0.68	0.50	62.40	0.30	6.54		80.08	91.60	0.106
2	48	4.18	1.48	53.10	6.79	0.50	0.50	58.00	0.50	6.30		74.43	92.78	0.101
3	48	3.98	1.50	55.60	7.06	0.50	0.50	59.00	0.50	6.40		74.72	90.65	0.102
4	54	3.88	1.41	50.60	6.47	0.50	0.50	58.00	0.20	6.00		73.43	92.74	0.090
5	110	4.43	1.16	50.00	6.16	0.80	0.80	55.00	0.30	5.80		70.58	94.16	0.067
6	50	4.40	1.74	42.60	6.00	0.60	0.30	51.00	0.70	5.80		65.45	96.67	0.051
7	91	4.30	1.86	45.00	6.36	0.50	0.30	49.00	0.40	5.38		62.88	84.59	0.027
8	77	3.80	1.18	43.50	5.53	-	0.20	52.00	0.50	5.70		66.73	97.92	0.023
Average*	73	4.17	1.59	43.70	5.96	-	-	50.70	0.56	5.63		65.02	92.76	0.033

*t*=fermentation time, *V*_B_=volume of the bioreactor, *φ*(ethanol)_i_ and *φ*(ethanol)_f_=initial and final volume fraction of ethanol, *γ*_i_=initial acidity, *φ*_AAi_ and *φ*_AAf_=initial and final volume fraction acetic acid in the broth, *V*_A_=volume of fermented broth (alcohol) added to the fermentor, *V*_R_=volume of the removed acetic acid, *γ*_f_=final acidity, *Y*_AA_=yield of acetic acid, *Y*_AA_(broth)=total yield of acetic acid in the broth, *P*_AA_=productivity. *Average value of the last three cycles

**Table 2:** Physicochemical characterisation of the soybean molasses vinegar

Component	Value
Acidity/(g/100 mL)	5.07±0.06
*m*(residual ethanol)/*V*(vinegar)%	0.34±0.02
*m*(residual sugar)/*V*(vinegar)/%	7.86±0.12
*m*(dry extract)/*V*(vinegar)%	14.67±0.07
*m*(ash)/*V*(vinegar)/%	2.27±0.10
*ρ*_relative_/(g/cm^3^)	1.02±0.00

The average time required for the acetification of soybean molasses (mean of the last three cycles) was 73 h. Among these three fermentation cycles (which contained only the fermented broth and ethanol from soybean molasses), the shortest fermentation time was 50 h (sixth cycle) and the longest was 91 h (seventh cycle). The highest acidity level and *Y*_AA_ (52.0 g/L and 66.7%, respectively) were reached after 77 h (eighth cycle). Spinosa *et al*. (*8*) produced vinegar from rice wine in a submerged fermentation and evaluated 10 fermentation cycles. During this process, the wine from alcohol fermentation contained 62.80 g/L ethanol, which produced a vinegar with an acetic acid content 65.80 g/L, *Y*_AA_=86.00% and 0.70 g/L acetic acid yield. In another work that aimed to obtain a vinegar from banana pulp, Tanaka *et al*. (*20*) recorded a maximal concentration of acetic acid ranging from 59.0 to 40.0 g/L, with an average of 49.7 g/L. At the end of our fermentation, the acidity, *Y*_AA_ and productivity were 50.70 g/L, 65.02% and 0.033 g/(L·h), respectively, on average, over the last three cycles.

The acetic acid production by the oxidation of ethanol is an exothermic reaction that requires oxygen. Consequently, the fermentation efficiency is highly related to the aeration and the air dispersion rates inside the reactor. The low productivity (*P*_AA_) observed in this work may also be associated with the non-homogeneous air distribution inside the fermentor. Other authors observed the same occurrence (*8*, *12*). Another problem that can cause a low acetic acid yield is the evaporation of ethanol. This loss occurs during fermentation and can reach up to 10–30% of the stoichiometric yield, depending on the temperature. A study of open, semi-open and closed acetic acid fermentor concluded that closed acetic acid fermentor is more appropriate for industry because of lower loss by evaporation (*14*).

### Characterisation of the vinegar

The acetic acid fermentation product of soybean molasses contained, on average, 5.07% (*m*/*V*) acetic acid, 0.17% (*m*/*V*) residual ethanol, 7.86% (*m*/*V*) dry extract, 2.27% (*m*/*V*) ash, and had a density of 1.023 g/cm^3^ (Table 2). According to the legislation of some countries, like Brazil, the minimum acidity levels required for the commercialisation of vinegar is 40 g/L (*8*). In this context, the acetic acid fermentation product obtained during this project conforms to the standards.

Different alcohol (ethanol) and acetic acid (vinegar) fermentation products with unique chemical characteristics can be obtained from different raw materials. For this reason, we analysed the contents of the total phenolic compounds and isoflavones of the products obtained from soybean molasses fermented with alcohol (ethanol) and acetic acid (vinegar) (Table 3). High concentrations of total phenolic compounds were observed in soybean molasses, ethanol and vinegar that were 1.52-, 61.0- and 53.9-fold higher than the total isoflavone content, respectively. Total phenolic content of the alcoholic (ethanol) and acetic acid (vinegar) fermentation products from soybean molasses decreased by 3.49 and 35.69%, respectively. However, when the alcohol fermentation was extended into the acetic acid fermentation, the total phenolic content decreased to 37.86%. The high concentration of total phenolic compounds of the ethanol and vinegar derived from soybean molasses might have been caused by the retention of some of these compounds during fermentation. Specifically, during alcohol fermentation new compounds could have been generated as a result of the presence of microorganisms (*21*).

**Table 3:** Contents of total phenolics (in mg GAE per 100 mL) and isoflavones (in mg per 100 mL on wet mass basis) in soybean molasses, wine (alcohol fermentation) and vinegar (acetic acid fermentation)

Fermentation product	Soybean molasses	Wine	Vinegar
Total phenolics	625±59	647±10	402±4
6″-*O*-Malonyl-daidzin	12.0±0.6	n.d.	n.d.
6″-*O*-Malonyl-genistin	10.4±0.3	n.d.	n.d.
Daidzin	125.8±2.0	n.d.	n.d.
Genistin	186.2±9.3	3.8±0.4	3.0±0.2
Glycitin	29.8±1.0	4.6±1.0	3.2±0.2
Daidzein	22.4±1.1	2.2±0.4	1.29±0.06
Genistein	25.2±0.6	n.d.	n.d.
Total isoflavones	411.8±14.0	10.6±1.8	7.5±0.5

GAE=gallic acid equivalents, n.d.=not detected

The total isoflavone content of soybean molasses (Table 3) was high, whereas during the alcohol and acetic acid fermentations, the content decreased to 38.8 and 55.64%, respectively. The total isoflavone content decreased only 1.42 times as the alcohol fermentation progressed into the acetic acid fermentation. In soybean molasses, the predominant forms of isoflavones were β-glycosides, aglycones and malonylglycosides at 83.01, 11.55 and 5.43%, respectively, and no acetylglycosides were detected. This result is interesting because these values report a very different isoflavone profile compared to soybean grain. Moreover, the absence of acetylglycoside isoflavones indicates that soybean molasses had not undergone any high thermal treatment during its production.

The predominant isoflavones of the 18 soybean cultivars investigated by Ribeiro *et al*. (*22*) were malonyldaidzin and malonylgenistin and together they accounted for 67.0% of the total isoflavone content. In the same study, there were 31.0% β-glycosides and 2.0% aglycones among total isoflavones, whereas the acetylated form was not detected. When heat-treated in an oven at 200 °C for 20 min, whole soybean meal showed an altered isoflavone content and a high conversion rate of malonylglycosides to acetylglycosides, β-glycosides and aglycones (*23*). Malonylgenistin has been described as the predominant isoflavone in soybean, and it can be converted to acetylgenestin or genistin after drying or hot-water extraction, respectively. Humid heat is more effective than dry heat to convert and degrade the isoflavones mentioned above (*24*). The contents of 6′′-*O*-malonyl-daidzin and 6′′-*O*-malonyl-genistin were low in soybean molasses (Table 3), and after the alcoholic and acetic acid fermentation, these two forms were no longer detectable. Moreover, no β-glycosides (daidzin) or aglycones (genistein) could be detected in the ethanol and vinegar produced during our study. Instead, β-glycosides (genistin and glycitin) and aglycones (daidzein) were the only forms of isoflavones present in both fermented products, at low concentrations. The low amounts or lack of detection of isoflavones in ethanol and vinegar was probably caused by the presence of yeasts, which may utilise certain cell wall phenolic compounds, as observed by Razmkhab *et al*. (*25*). The defatted soybean meal fermented in the presence of *Monascus purpureus* or *Aspergillus oryzae* at an adequate water/ethanol ratio was considered the ideal method to simultaneously extract phenolic compounds and isoflavones with high antioxidant activities (*26*).

There is a growing interest among consumers and industries for the production of antioxidant-rich soy products (*27*). In this study, we obtained alcohol- and acetic acid-fermented products from soybean molasses with a unique chemical composition of total phenolic compounds and isoflavones. In particular, the presence of aglycones (daidzein) in our products is especially remarkable, as these compounds are deemed highly beneficial to human health.

## CONCLUSION

The current study demonstrated that soybean molasses could be used to produce vinegar. Moreover, based on our results, each tonne of soybean molasses can produce an estimated 3460 L of vinegar, with an acetic acid concentration of 40 g/L, which is equivalent to 139 kg of acetic acid and satisfies the minimum concentration required by the legislation on vinegar production. Additional studies should be carried out to improve the fermentation conditions and thereby increase productivity, while simultaneously reducing the extent of the acetic acid fermentation. Furthermore, for soybean molasses to be proposed for human consumption, a profile of the anti-nutritional factors and some sensorial studies still need to be performed.
